# Brain state transition analysis using ultra-fast fMRI differentiates MCI from cognitively normal controls

**DOI:** 10.3389/fnins.2022.975305

**Published:** 2022-09-28

**Authors:** William C. Palmer, Sung Min Park, Swati Rane Levendovszky

**Affiliations:** Department of Radiology, University of Washington, Seattle, WA, United States

**Keywords:** ultrafast fMRI, Alzheimer’s disease, mild cognitive impairment, dynamic functional connectivity, BOLD (blood oxygenation level dependent) signal

## Abstract

**Purpose:**

Conventional resting-state fMRI studies indicate that many cortical and subcortical regions have altered function in Alzheimer’s disease (AD) but the nature of this alteration has remained unclear. Ultrafast fMRIs with sub-second acquisition times have the potential to improve signal contrast and enable advanced analyses to understand temporal interactions between brain regions as opposed to spatial interactions. In this work, we leverage such fast fMRI acquisitions from Alzheimer’s disease Neuroimaging Initiative to understand temporal differences in the interactions between resting-state networks in 55 older adults with mild cognitive impairment (MCI) and 50 cognitively normal healthy controls.

**Methods:**

We used a sliding window approach followed by k-means clustering. At each window, we computed connectivity i.e., correlations within and across the regions of the default mode, salience, dorsal attention, and frontoparietal network. Visual and somatosensory networks were excluded due to their lack of association with AD. Using the Davies–Bouldin index, we identified clusters of windows with distinct connectivity patterns, also referred to as brain states. The fMRI time courses were converted into time courses depicting brain state transition. From these state time course, we calculated the dwell time for each state i.e., how long a participant spent in each state. We determined how likely a participant transitioned between brain states. Both metrics were compared between MCI participants and controls using a false discovery rate correction of multiple comparisons at a threshold of. 0.05.

**Results:**

We identified 8 distinct brain states representing connectivity within and between the resting state networks. We identified three transitions that were different between controls and MCI, all involving transitions in connectivity between frontoparietal, dorsal attention, and default mode networks (p<0.04).

**Conclusion:**

We show that ultra-fast fMRI paired with dynamic functional connectivity analysis allows us to capture temporal transitions between brain states. Most changes were associated with transitions between the frontoparietal and dorsal attention networks connectivity and their interaction with the default mode network. Although future work needs to validate these findings, the brain networks identified in our work are known to interact with each other and play an important role in cognitive function and memory impairment in AD.

## Introduction

Alzheimer’s disease (AD) is currently the leading cause of dementia and occurs due to the accumulation of amyloid-β (Aβ) and hyper-phosphorylated tau, a process that begins years before clinical manifestation (Price and Morris, 1999; Morris et al., 2010; Scheltens et al., 2021). Much work has focused on reducing amyloid accumulation by reducing amyloid deposition and enhancing its clearance. Considerable effort has gone into uncovering efficacious interventions and treatment strategies, but their failure behooves us to discover alternative pathological processes, which may provide critical opportunities to slow or prevent disease progression at its early stages. For that reason, attention has shifted toward identifying biomarkers for earlier stages of the AD continuum such as prodromal disease stages, mild cognitive impairment (MCI), and early AD (Sperling et al., 2011). The key markers of AD pathology have been defined as the presence of Aβ and phosphorylated tau in the cerebrospinal fluid (Jack et al., 2018). Less invasive methods in the form of neuroimaging also play a critical role in the identification of AD (Márquez and Yassa, 2019). Magnetic resonance imaging (MRI) is unable to detect amyloid or tau deposition in the brain but contributes to our understanding of related disease processes in the AD brain.

In addition to structural MRI, further promise has been demonstrated by the emerging field of resting-state functional connectivity (rsFC) computed from low-frequency blood oxygenation level-dependent (BOLD) signal in the absence of a task (Lee et al., 2013). Spontaneous BOLD signals at rest (in the absence of an evoked response) were shown to be associated with neural origins and correlations between them revealed connectivity between different regions of the brain (Biswal et al., 1995). These resting state networks could represent the brain’s intrinsic organization (Fox et al., 2006; Seeley et al., 2007).

Differences in the functional connectivity (i.e., correlation) between the nodes of these resting state networks have been identified in a number of brain diseases including multiple stages of AD (Fox et al., 2006; Lee et al., 2013; Sheline and Raichle, 2013; Lin et al., 2018; Márquez and Yassa, 2019). In AD, these disruptions have been identified following PET identification of amyloid deposition, but preceding neurodegeneration making these disruptions a prime biomarker for early stages of the AD continuum (Sheline and Raichle, 2013). The most commonly implicated resting state network in MCI is the default mode network (DMN) (Sheline and Raichle, 2013; Lin et al., 2018). This is not surprising as the cortical areas that comprise the DMN show the first signs of abnormal amyloid deposition and AD pathology correspond with alterations in functional connectivity of the DMN (Brier et al., 2014; Palmqvist et al., 2017). Cognitive networks other than the DMN have also been implicated (Agosta et al., 2012; Brier et al., 2012). For example, anticorrelation between DMN and dorsal attention network (DAN) has been demonstrated to be significantly decreased in MCI compared to normal aging controls (Esposito et al., 2018). The salience network (Sal) and it’s interaction with DMN have also specifically been identified as disrupted in MCI (He et al., 2013; Zhan et al., 2016). The frontoparietal network (FPN) is responsible for many cognitive tasks of varying demands. It is shown to coordinate with the DMN, DAN, and Sal (Cole et al., 2013). In this work, we study the temporal dynamics of these four networks.

Most studies to-date utilize static rsFC analyses which assume that brain regions and their connections to each other over the span of an entire scan remain unchanged (Biswal et al., 1995; Lee et al., 2013). However, it has been demonstrated that connectivity between brain regions varies over time at the level of minutes to seconds in fMRI proving the dynamic nature of rsFC (Chang and Glover, 2010; Hutchison et al., 2013b). A number of novel approaches have emerged to leverage this dynamicity to better understand differences between older adults with neurodegenerative diseases such as AD and healthy controls (Preti et al., 2017; Filippi et al., 2019). Some studies have investigated dynamic functional connectivity differences in the AD continuum compared to control participants. These studies discovered consistent decreases in global metastability (strength of coordinated whole-brain oscillations) in AD and MCI individuals compared to controls, which could indicate that individuals on the AD continuum have a smaller set of functional configurations (Córdova-Palomera et al., 2017; Demirtaş et al., 2017). Others have used independent component analysis to first identify resting state networks and estimated connectivity between components. Using this approach Sendi et al. found that the middle frontal gyrus and inferior parietal lobule had altered connectivity longitudinally. Both are regions of the DMN. Dwell times corresponding to these states were also longer in normal compared to mild AD in this study (Sendi et al., 2021). Furthermore, these dynamic connectivity metrics have been demonstrated to provided increased predictive power when identifying those with early MCI (Wee et al., 2016).

Our study seeks to build on these studies by leveraging ultrafast acquisition fMRI images collect by the Alzheimer’s Disease Neuroimaging Initiative (ADNI 3.0) and dynamic state analysis technique (Allen et al., 2014; Vergara et al., 2020). This technique identifies characteristic brain connectivity patterns or states by clustering sliding-window connectivity matrices. Differences in dynamics are captured by metrics such as dwell time, which measures the amount of time the brain remains in one state, and transition probability, which is the chance the brain moves from one state to another. We hypothesized that we would observe differences in these metrics of dynamic states involving the cognitive resting state networks (DMN, Sal, DAN, and FPN) that distinguish older adults with cognitive impairment from healthy controls.

## Materials and methods

### Participants

The data used for this study was collected from the ADNI database^1^. Specifically, a subset of participants was selected that had undergone ADNI 3.0 advanced protocol scanning which included sub-second functional scans. Out of the 107 participants available, two were excluded due to a missing structural image or insufficient data. In these analyses those with the early amnestic MCI, late MCI, subjective memory concerns, or mild Alzheimer’s disease dementia clinical labels were combined into one group which will be referred to as MCI (*n* = 50) in this study. Cognitively normal subjects were used as healthy controls (*n* = 55). Mini-Mental State Examination (MMSE) scores were also collected for all participants as a measurement of cognitive functioning with low scores reflecting higher cognitive impairment (Tombaugh and McIntyre, 1992).

### Imaging

All images were acquired on 3T scanners across 12 sites. Anatomical T1 weighted scans were 3D MPRAGE sagittal acquisition. The spatial resolution was 1 × 1 × 1 mm^3^, repetition time (TR)/echo time (TE) was 2,300 ms/min full echo, and inversion time (TI) was 900 ms. The BOLD functional axial scan was a whole-brain echo planar acquisition. The resolution was 2.5 × 2.5 × 2.5 mm^3^, TR/TE was 600/30 ms, flip angle was 53°, and acquisition matrix was 220 × 220 × 160 mm^3^. During the scan, 976 volumes were collected.

### Analysis

#### Preprocessing

Preprocessing was completed using a combination of FSL (5.0), AFNI (16.0.11) and the CompCor algorithm (niak-boss-0.13.0) (Cox, 1996; Behzadi et al., 2007; Jenkinson et al., 2012). Anatomical images were skull-stripped and segmented into gray matter (GM), white matter (WM), and cerebrospinal fluid (CSF). Functional image processing began with the removal of the first five volumes. The remaining volumes were then motion-corrected, and baseline drift was removed using a 0.01 Hz high pass filter in FSL. Despiking was conducted with AFNI to remove outlier motion. Eroded WM and CSF masks created from the anatomical images were registered to the functional images. The first five principal components, which approximate physiological noise, were generated from signals extracted with these masks and regressed out according to the CompCor algorithm (Behzadi et al., 2007). Finally, volumes were variance normalized, registered to 2 mm MNI space, and smoothed with a full width half maximum kernel of 6 mm.

#### Regions of interest

Regions of interest (ROI) consisted of four large scale cortical resting state networks which included DMN, Sal, DAN, and FPN as delineated by Yeo’s 7 network atlas (Yeo et al., 2011). The four networks were divided into 32 non-overlapping regions according to FSL’s Harvard-Oxford structural atlas. The mean time course was calculated for each of these ROIs.

#### Sliding-window dynamic clustering

The dynamic clustering analysis followed a method previously defined by Allen et al. (2014). In short, sliding-window series were created from the mean ROI time courses with a stride length of 1 volume and windows containing 50 volumes (30 s of data). Connectivity matrices were constructed for each of these windows with correlation as the connectivity metric. These symmetric connectivity matrices were transformed into vectors using elements of the off diagonal lower triangular matrix. The transformation resulted in a 496 by 921 matrix for each participant. These matrices were then harmonized with ComBat to reduce site-related variability (Yu et al., 2018). ComBat (Combining Batches), originally used in genomic studies, is a well-established data-driven harmonization method applied to features (such as connectivity values) extracted from imaging data (such as resting-state fMRI). It uses a Bayesian estimation approach to reduce site-related systematic variability while retaining biological variability. Site information is encoded as a variable where variability needs to be minimized, while diagnosis, age, and gender are designated as variables of interest where variability needs to be preserved. It has been successfully used in many multi-modal MRI studies.

**FIGURE 1:**
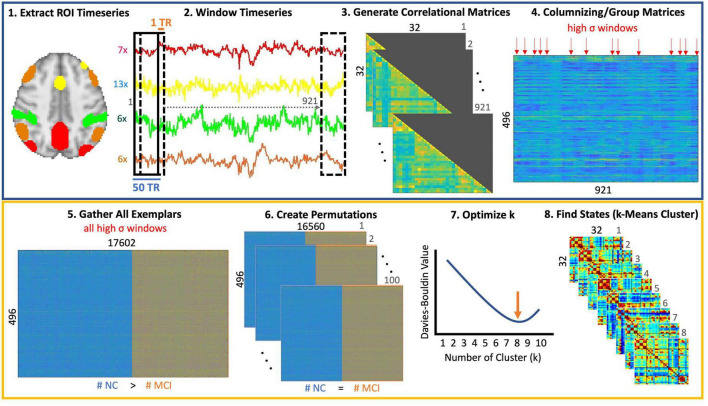
Step-by-step brain state identification. (1) The preprocessed fMRI time series data was registered to MNI space. The resting-state networks masks for the default mode network (DMN), dorsal attention network (DAN), Salience network (Sal), and the Frontoparietal Network (FPN) were chosen from the Yeo atlas. Individual regions within each mask were delineated by intersecting these masks with regions of the Harvard Oxford Cortical and Subcortical Atlas in FSL. Seven regions (frontal pole, superior and inferior lateral occipital, juxtapositional lobule, cingulate, precuneus, cuneus) were identified for the DMN. The Salience network comprised of 13 regions (superior frontal, middle frontal, temporal pole, temporal pole, anterior and posterior supramarginal gyrus, subcallosum, cuneus, parietal operculum, planum polare, Heschl’s gyrus, supracalcarine cortex), the DAN had 6 regions (superior parietal, anterior and posterior supramarginal gyrus, angular gyrus, inferior lateral occipital, cuneus), and the FPN consisted of 6 regions (insula, middle frontal, inferior frontal, angular gyrus, inferior and superior lateral occipital) as well. (2) A sliding window of 50 TRs was used in this work with a stride length of 1 TR. (3) For each window, a correlation matrix was calculated and only the lower triangular matrix was retained due to the symmetric nature of connectivity matrices. (4) Since the window was only shifted by 1TR, there are likely redundant windows where connectivity patterns do not change significantly between neighboring windows. To reduce this redundancy, only windows, which were the most different from the preceding and following windows were considered. This was performed by retaining those windows with local maxima in variance (indicated by red arrows). (5) All such windows (i.e., exemplars) were collected for all participants. (6) Since the number of exemplars in controls was greater than those in the MCI group, we created permutations such that the number of exemplars in both groups was identical. (7) K-mean clustering was performed for each permutation and the Davies Bouldin value was calculated at each permutation to identify the optimal number of clusters (i.e., with the lowest Davies Bouldin values). (8) Finally, k-means clustering was performed on the entire dataset using the optimal value of 8 clusters.

K-means clustering was performed on a subset of these windows with the highest variance (i.e., windows most dissimilar from their immediate neighbors on both sides), which will be referred to as the exemplar windows. The number of these exemplars was not equally distributed between the MCI and control groups, so the optimal number of states was determined using 100 permutations consisting of randomly sampled exemplars from MCI and control participants. The number of exemplars for both MCI and control participants was equal in each permutation (Leonardi and Ville, 2014). Based on previous work by Vergara et al. (2020), Davies-Bouldin values were used to determine the optimal number of distinct states. We used the correlation distance metric to uncover relative connectivity patterns between regions. The number of clusters with lowest Davies Bouldin value was selected as the optimal number of clusters. Once the optimal number of states was identified, k-means clustering was performed on all exemplars, and the resulting states were used to classify all connectivity vectors. A diagram of this processing is included in Figure 1.

#### Group differences

Dwell time and transition probabilities were calculated for each participant’s time series. Dwell time is defined as the number of windows that a participant remained in a state (where S_t_ = S_t+1_). Transition probabilities represent the probability that a participant would progress from one state to another. A generalized linear model was then used to identify significant differences in these metrics between MCI and control groups while correcting for sex and age as confounding variables. For instance, Dwell time ∼ Age + Sex + Diagnosis. A false-discovery-rate correction for multiple comparisons was applied at a threshold of 0.05 considering 8 comparison, one corresponding to each state.

## Results

### Clinical and demographic data

Fifty MCI participants and fifty-five control participants were included in the analysis. Table 1 lists participant information. No significant difference was observed in age or sex distribution. The MCI participants (26.3 ± 5.4) scored significantly (*p* = 0.0008) lower than controls (28.9 ± 1.2) on the MMSE. This was expected since the two groups are differentiated by cognitive impairment.

**TABLE 1:** Participant demographics.

Description	Controls	Mild cognitive impairment (MCI)
Sample Size	55	50
Sex	29 M	20 M
Age (years)	74.4 ± 7.8	75.0 ± 7.9
MMSE	28.9 ± 1.2	26.3 ± 5.4*

*p = 0.0008.

### Identified states

**FIGURE 2:**
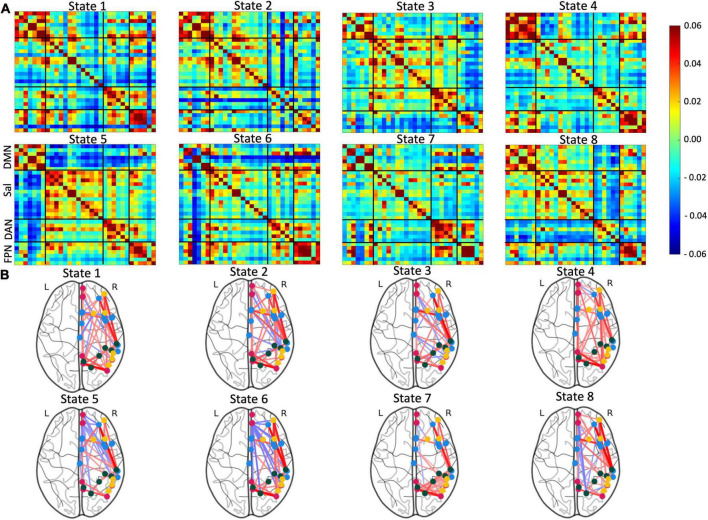
Visualization of the eight identified brain states. **(A)** Connectivity matrices of the identified brain states with Fisher Z and L1-normalized correlations. **(B)** Corresponding glass brain diagrams of the brain states. Red connections indicate positive correlations and blue connections indicate negative correlations. For full, glass brain plots see Supplementary Figure 2.

The optimal state number (k) was determined by the lowest Davies-Bouldin value. In our case, the lowest value was 4.5836 ± 0.0211, which was calculated with eight states. Clustering with seven and nine states had similar values but were more variable (*k* = 7: 4.5841 ± 0.0557, *k* = 9: 4.6058 ± 0.0489; Supplementary Figure 1). Therefore, the final clustering was performed with eight states (Figure 2 and Supplementary Figure 2 for full brain diagrams).

The first state was characterized by high positive correlation within the DMN and FPN and moderate positive connectivity in the DAN and some positive connectivity between these three networks. There also existed strong negative connectivity between the superior frontal gyrus FPN node and all other areas. The second state has strong positive connectivity in the DMN along with modest positive connectivity in Sal and FPN. The posterior supramarginal gyrus and postcentral gyrus components of DAN showed consistent negative connectivity with other nodes, particularly those outside DAN. Similar to State 1, the superior frontal gyrus continued to have negative connectivity to other nodes, however, to a lesser extent. State 3 displayed positive connectivity within all analyzed networks (DMN, Sal, DAN, and FPN). There was also some positive connectivity between Sal and DAN. Furthermore, the FPN had relatively consistent negative connectivity with the other networks. The fourth state displayed some of the highest positive connectivity within the DMN and FPN. There was also moderate positive connectivity between these two networks. State 5 was defined by moderate connectivity within all four networks. Sal, DAN, and FPN showed positive connectivity to each other, but all three were mostly anticorrelated to DMN. The sixth state has strong positive connectivity within the FPN and mixed connectivity between and within other networks. The frontal and cingulate gyrus of the DMN showed high positive connectivity with each other and strong negative connectivity with all other nodes. State 7 was characterized by positive connectivity within and between DAN and FPN. Some nodes within the DMN also demonstrated a high positive correlation. The final state, eight, had modest positive connectivity within and between networks except for high positive connectivity within the FPN. Additionally, the DAN demonstrated modest anticorrelation with all other networks. Note that all correlation matrices with converted to Z-core using Fisher transformation and then l1-normalized to Gaussianize the data.

### Differences between mild cognitive impairment participants and controls

**FIGURE 3:**
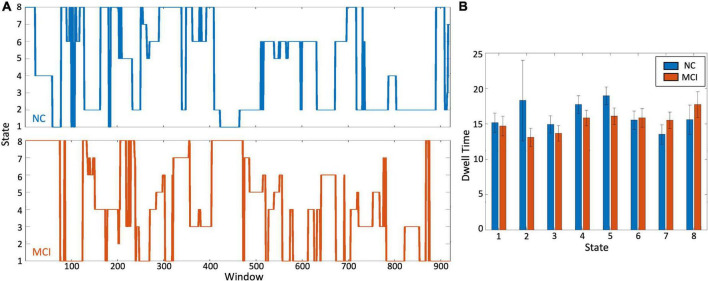
Differences in brain state dwell time. **(A)** Representative time course of window transitions over the duration of the scan for a healthy control participant in blue and an MCI participant in orange. There is no one-to-one correspondence between the two time-courses. **(B)** Comparing the dwell time i.e., time (in terms of number of windows) spent in each state, there was no significant difference between the two groups. Table 2 shows the actual dwell times.

All sliding window connectivity vectors were assigned to one of the eight states for all participants. This generated a timeseries tracing the states the brain traversed during their scan. Example MCI and control state timeseries are plotted in Figure 3A. The dwell time and transition probabilities were calculated from these state-matched timeseries.

**TABLE 2:** Comparison of Dwell times between controls and mild cognitive impairment (MCI)^1^.

State	Controls (Mean ± Std. Dev)	MCI, (Mean ± Std. Dev)	*p*
1	15 ± 10	15 ± 10	0.6
2	13 ± 9	18 ± 42	0.3
3	14 ± 8	15 ± 9	0.3
4	16 ± 8	18 ± 9	0.4
5	16 ± 8	19 ± 9	0.1
6	16 ± 9	15 ± 10	0.6
7	15 ± 8	13 ± 10	0.3
8	18 ± 13	16 ± 15	0.5

^1^Note that the dwell times are in number of windows that the brain spends each state in. Each window in this study is 30 s long and separated by the adjacent window by 1 TR I.e., 0.6 s. Therefore, a dwell time of 15 would corresponds to 39 s.

When investigating differences between MCI participants and controls, no significant differences were detected between groups in dwell time for all eight states (Figure 3B and Table 2). The difference that was closest to approaching significance was dwell time in state 5. Controls tended to spend more time in this state than MCI participants (*p* = 0.1). The *p*-value for all other differences were substantially greater (state 1: *p* = 0.6, state 2: *p* = 0.3, state 3: *p* = 0.3, state 4: *p* = 0.4, state 6: *p* = 0.6, state 7: *p* = 0.3, state 8: *p* = 0.5).

**FIGURE 4:**
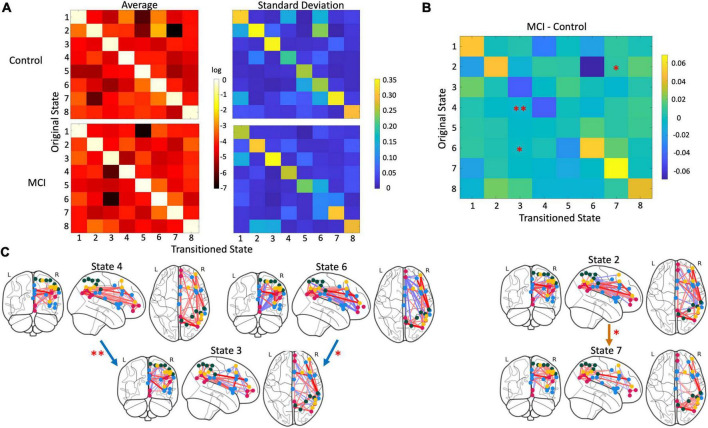
Differences in state transition probability. **(A)** Matrices with a mean (left) and standard deviation (right) of the log-transformed transition probability i.e., how likely a participant moved from one state to another, with controls above and MCI participants below. **(B)** Three transitions were significantly different between groups: The transition from state 4 to state 3 (*p* = 0.002), state 6 to state 3 (*p* = 0.009), and state 2 to state 7 (*p* = 0.04). MCI participants were less likely to switch from state 4 to state 3 and from state 6 to 3 than control participants. But MCI participants were more likely switch from state 2 to 7 compared to controls. **(C)** Glass brain plots of significant transitions with blue arrows indicating transitions controls were more likely to take and orange arrows indicating transitions MCI participants were more likely to undergo.

On the other hand, three transition probabilities were significantly different between the MCI and control groups (Figure 4). MCI participants were significantly less likely to transition from state 4 to state 3 than controls (*p* = 0.002). Similarly, compared to controls, older adults with MCI had a lower probability of moving from state 6 to state 3 (*p* = 0.009). However, MCI participants were more likely to transition from state 2 to state 7 than controls (*p* = 0.04).

## Discussion

This study shows significant differences in state transition probabilities between MCI participants and controls but no significant differences in dwell time when investigating connectivity within and between the DMN, Sal, DAN, and FPN. The most important results demonstrated that controls were more likely to transition from states with high positive connectivity within the FPN to a state with moderate positive intra-network connectivity and internetwork connectivity characterized by anticorrelation between the FPN and all other networks and some positive coordination between Sal and DAN. The high positive connectivity within the FPN immediately preceding segregated activity in DAN/Sal and DMN with anticorrelation to FPN could reflect the FPN’s role in task switching and its deficit in AD continuum disorders.

Since its discovery, the FPN has been hypothesized to mediate the relationship between DMN and DAN because of its anatomical interposition between the two (Vincent et al., 2008). The ability to move between these contrasting networks is critical because the DMN is responsible for internally motivated states. At the same time, the DAN is responsible for executive attention to external stimuli (Greicius et al., 2003; Fox et al., 2006; Seeley et al., 2007; Buckner et al., 2008). Following this, support for the executive task switching role of the FPN has continued to grow. Cole et al. (2013) demonstrated that the FPN has the most variable connectivity to other networks across different tasks. Its connectivity to other networks can predict the type of task performed. Decreased FPN white matter integrity has also been correlated to age-related slowing of task-switching performance (Gold et al., 2010). White matter hyperintensities in the FPN circuit have been associated with declines in executive functioning in individuals with MCI (Jacobs et al., 2012). This evidence points to the FPN’s role in flexibly changing network configurations to match present needs (Marek and Dosenbach, 2018). In AD continuum disorders, abnormal interactions between the DMN and DAN, which the FPN mediates, have been indicated. Brier and team found that increases in cognitive status severity corresponded to decreased anticorrelation between DMN and DAN, similar to that seen in state 8 in our study (Brier et al., 2012). Additionally, DMN and DAN negative connectivity was reduced in individuals with MCI compared to elderly controls (Esposito et al., 2018). Furthermore, a meta-analysis study has directly implicated the FPN in MCI individuals finding hypoactivation in the DMN and FPN compared to controls (Li et al., 2014). A lack of the FPN’s ability to drive diverse normal network interactions could also explain a decrease in the dynamic repertoire in MCI (Córdova-Palomera et al., 2017; Demirtaş et al., 2017).

Our other result indicated that MCI participants were more likely to move away from state 2 (a state characterized by strong positive connectivity within the DMN and some positive connectivity between DMN and Sal) to state 7, with primarily high positive connectivity within and between the DAN and FPN. The salience network is involved in identifying salient stimuli and can flexibly interact with other networks given the desired cognitive state (Seeley et al., 2007; Chen et al., 2016). Multiple studies have found that individuals with MCI have decreased connectivity between Sal and DMN nodes (He et al., 2013; Zhan et al., 2016). Interestingly in cognitively normal participants with AD pathology, amyloid-positive individuals with high Tau-PET levels had hypoconnectivity between Sal and DMN (Schultz et al., 2017). This decrease in connectivity could be explained by MCI participants moving from states with positive connectivity between Sal and DMN and high connectivity within these networks to states with no coordination between and drastically decreased connectivity within Sal and DMN.

We leverage ultrafast fMRI to understand *temporal changes* in connectivity within and between resting state networks. Static fMRI analyses do not allow us to evaluate such temporal changes within and between network changes. For our purpose, to understand the temporal evolution of functional connectivity during the scan duration we used the sliding window approach. Ideal window size to understand brain dynamics is yet unknown but typical recommendations are 20–120 s for such approaches (Hutchison et al., 2013a; Leonardi and Van De Ville, 2015). Jones et al., showed that brain states in a population of older adults with and without AD, typically stabilize around 27 s (Jones et al., 2012). Typical fMRI with TR around 2 s then would have 15 volumes per window as opposed to 50 volumes in our data. While the study would be feasible at typical fMRI of TR = 2 s, the ultra-fast fMRI provides the advantage of larger data sample per window and effect sizes. Additionally, our approach of bootstrapping and reselecting windows mitigates the issue of selecting only spurious changes that may occur at shorter time scales as observed by some studies (Leonardi and Van De Ville, 2015).

Furthermore, it is important to note that different TRs will be sensitive to different temporal dynamics that are dependent on neuronal populations, sizes, and regions that make up local brain network or systems (Buzsáki et al., 2013). Brain oscillations span a large range 0.01–10 Hz requiring sampling intervals or TRs of 100 ms to almost 10 s. Typical TRs of 2–3 s allow us to capture slow hemodynamic fluctuations while faster TRs (< 1 s) allows us to capture fluctuations that are more reflective of neuronal dynamics. This is clearly shown by Zhang et al. in their recent work (Yang and Lewis, 2021). We analyzed the ultrafast fMRI data by resampling the data at TR = 2.4 s, with each window now containing only 13 time-points instead of 50. The connectivity matrices are shown in Supplementary Figure 3. Of note, with the Davies Bouldin criteria, even with 20 clusters, no optimal number of clusters/states was detected. Restricting the states to 8, similar to that of the ultrafast acquisition, we found similar brain states but no significant transitions or differences in dwell times. Although distinct in the information obtained, we also evaluated static connectivity in the two group (Supplementary Figure 4). No significant differences were observed. As before, for both comparisons, a FDR correction of 0.05 was used.

While our results demonstrate correspondence with knowledge from static resting-state network functional connectivity in MCI, other studies are still necessary to confirm differences in state transition probability. Subsequent studies should specifically focus on using newer dynamic techniques and measurements of connectivity. Sliding-window k-means clustering techniques are often criticized for the inherent high correlation between windows (smearing of the BOLD time series) and arbitrary choice of metrics for optimizing the number of distinct states. We tried to mediate these issues by clustering using exemplars. This method simultaneously reduces data dimensionality while excluding similar consecutive windows from state clustering. Furthermore, we optimized clustering using the Davies-Bouldin metric that demonstrated a higher accuracy in dynamic clustering when compared to other metrics (Vergara et al., 2020). However, other techniques exist that avoid these issues, such as Hidden Markov Models (Taghia et al., 2017, 2018). Furthermore, alternative connectivity metrics other than correlation should be explored as correlation captures linear direct and indirect statistical dependencies and does not provide information about directionality. Other metrics that have been proposed as substitutes include mutual information, dynamic time warping, and unnormalized partial correlation (precision matrix) (Liégeois et al., 2020; Mohanty et al., 2020). New generative model-based connectivity metrics also provide directional information or the influence that one node has over another, such as Dynamic Causal Modeling and Mesoscale Individualized Neurodynamic (MINDy) modeling (Friston et al., 2003; Singh et al., 2020).

## Conclusion

We demonstrated that MCI participants significantly differed from controls in their transition probabilities between brain states involving DMN, Sal, DAN, and FPN. These results are consistent with findings in static functional connectivity while respecting the innate dynamicity of the brain. They also shed further light on static connectivity results, possibly demonstrating that the brain signature of MCI includes reduced FPN-enforced patterns of connectivity and increased transitions away from connectivity between Sal and DMN. However, these results must be investigated further using novel dynamic functional connectivity techniques. Our study supports the importance of dynamic functional connectivity to our understanding of brain dysfunction in MCI and its potential use as a novel biomarker.

## Data availability statement

Pubicly available datasets were analyzed in this study. This data can be found here: Alzheimer’s Disease Neuroimaging Initiative (ADNI): https://adni.loni.usc.edu/.

## Ethics statement

Ethical review and approval were not required for the study on human participants in accordance with the local legislation and institutional requirements. Written informed consent for participation was not required for this study in accordance with the national legislation and the institutional requirements.

## Author contributions

SL: conceptual design, implementation, data interpretation, and manuscript editing. WP: data analysis and manuscript writing. SP: data preprocessing and manuscript editing. All authors contributed to the article and approved the submitted version.

## Footnotes

1
https://adni.loni.usc.edu/

## Supplementary material

Supplementary Figure 1 Davies–Bouldin plot to choose the optimal number of clusters for a window size of 30 s at Tr = 0.6 s.

Supplementary Figure 2 Three-plane glass brain view of the eight brain states identified in this work. Red connections indicate positive correlations and blue connections indicate negative correlations.

Supplementary Figure 3 Connectivity matrices corresponding to the 8 states identified using TR = 0.6 s and using the same data but subsampled at TR = 2.4 s.

Supplementary Figure 4 Groupwise static connectivity between the same regions as the regions of the DMN, Sal, DAN, and FPN used for dynamic functional connectivity. No significant difference was observed between groups.
