# Supplementary material for: Brain state transition analysis using ultra-fast fMRI differentiates MCI from cognitively normal controls
Source: Front Neurosci. 2022 Sep 28;16:975305. doi: 10.3389/fnins.2022.975305 (PMC9555083; doi:10.3389/fnins.2022.975305)
Supplement: Supplementary file 1 [file Table_1.DOCX]

**Supplementary Materials:**

We performed identical analysis on the data by considering different window lengths 60, 90, and 120 and compared how many states could be uniquely identified and what brain states determined differences between MCI and controls. For the sake of uniformity in comparison and the lack of a deterministic number of clusters using the Davies Bouldin criteria, we used 8 clusters per window size.

Each window size visually shows similarities in the brain state connectivity patterns, with most changes at 120 s. However as expected, just like different TR values, each capture different dynamic properties as illustrated in the Table below.

The cluster matrices are shown below:


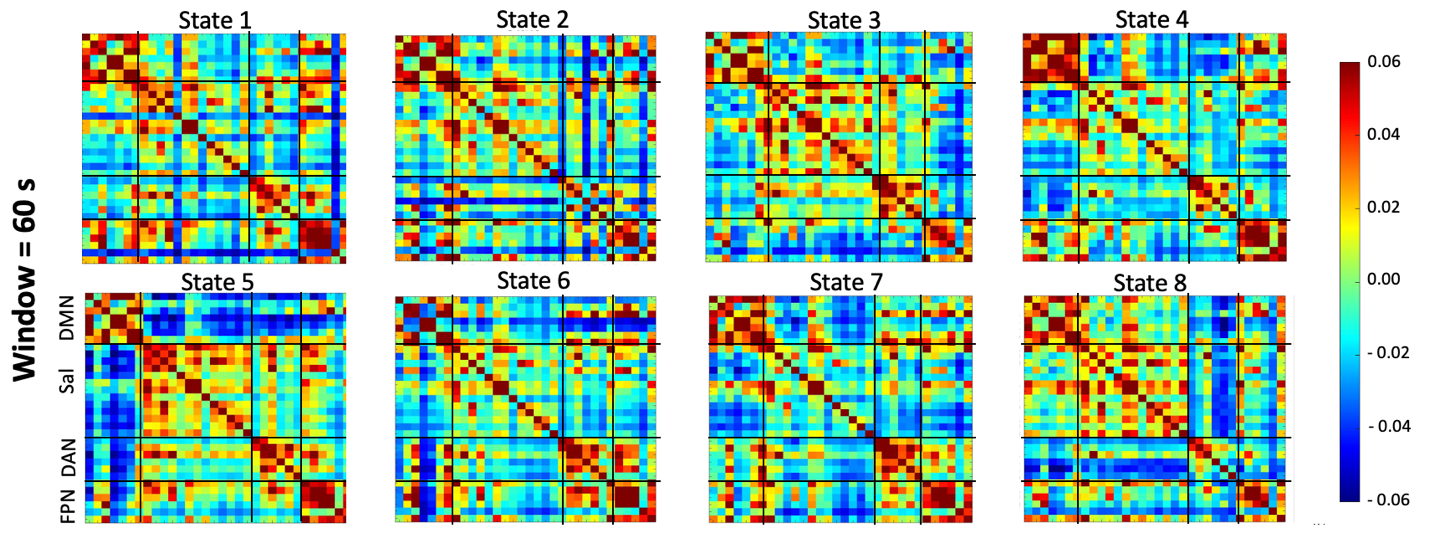


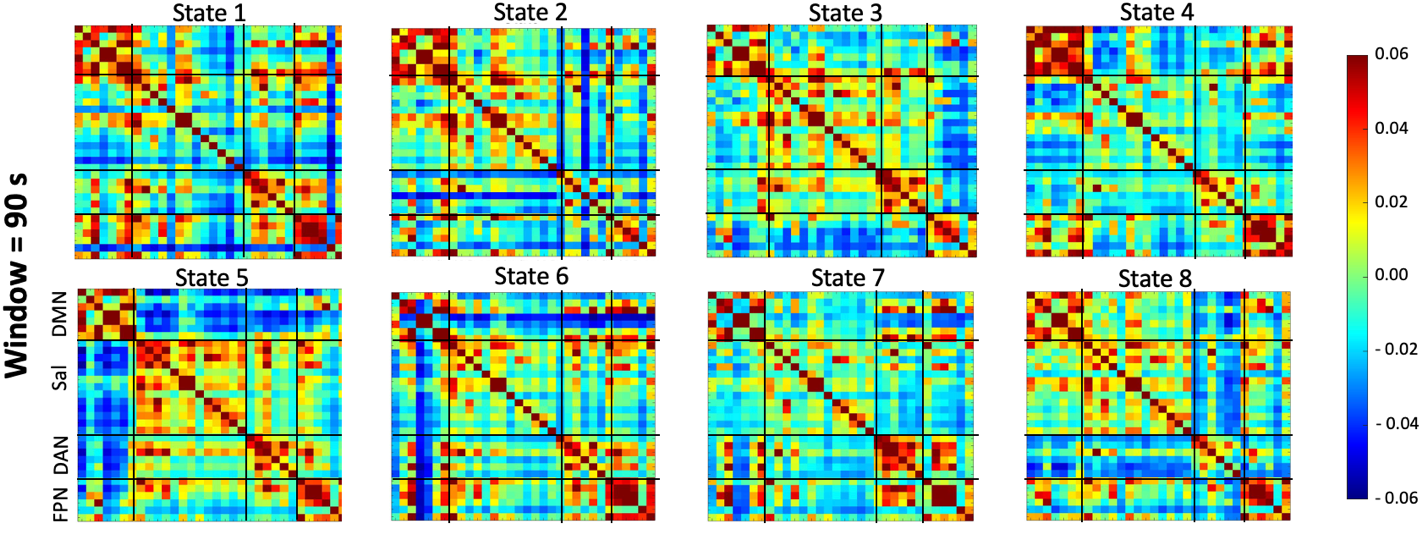


**
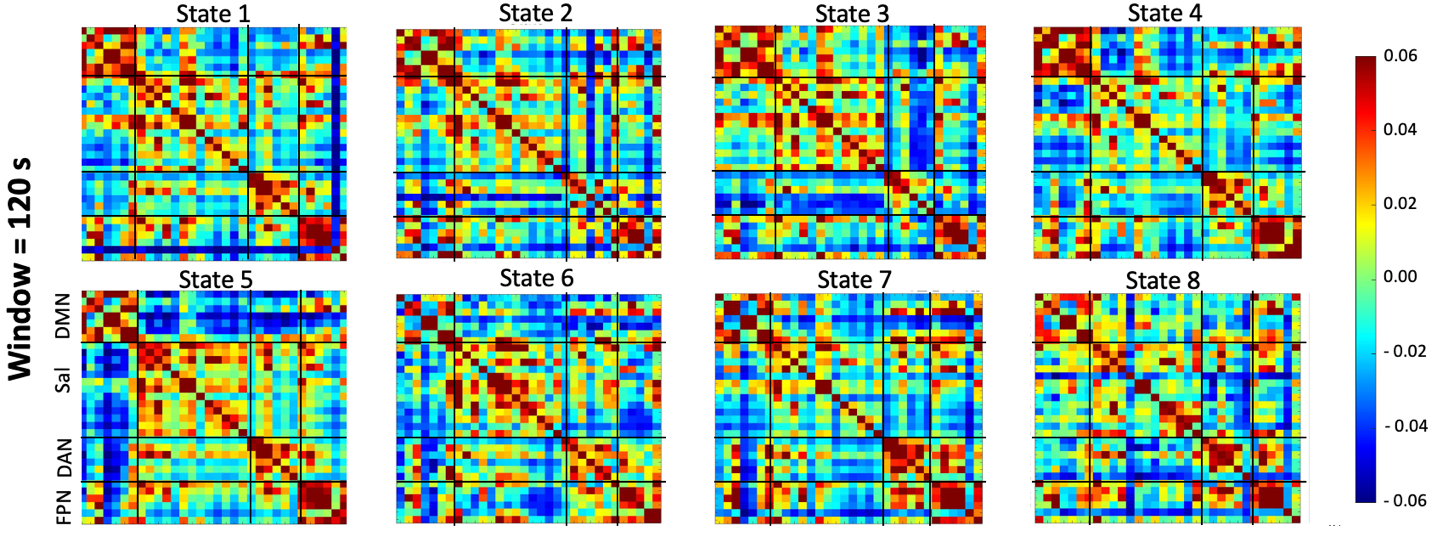
**

**Table 1: Comparison of different window lengths**

| Window length | Unique clusters per Davies Bouldin criterion | Significant differences in state transition between groups | Significant differences in dwell times between groups |
| --- | --- | --- | --- |
| 30 s | 8 | 4→3 (0.002)  6→3 (0.009)  2→7 (0.04) | None |
| 60 s | Continually decreasing value until n=20 clusters, No determinable cut-off | 2→6 (0.04) | None |
| 90 s | 8 | 4→2 (0.04)  3→2 (0.04)  1→7 (0.03)  8→7 (0.003)  6→7 (0.006) | None |
| 120 s | Continually decreasing value until 20 clusters, No determinable cut-off | None | None |
