# Supplementary figures and images for: Brain state transition analysis using ultra-fast fMRI differentiates MCI from cognitively normal controls
Source: Front Neurosci. 2022 Sep 28;16:975305. doi: 10.3389/fnins.2022.975305 (PMC9555083; doi:10.3389/fnins.2022.975305)

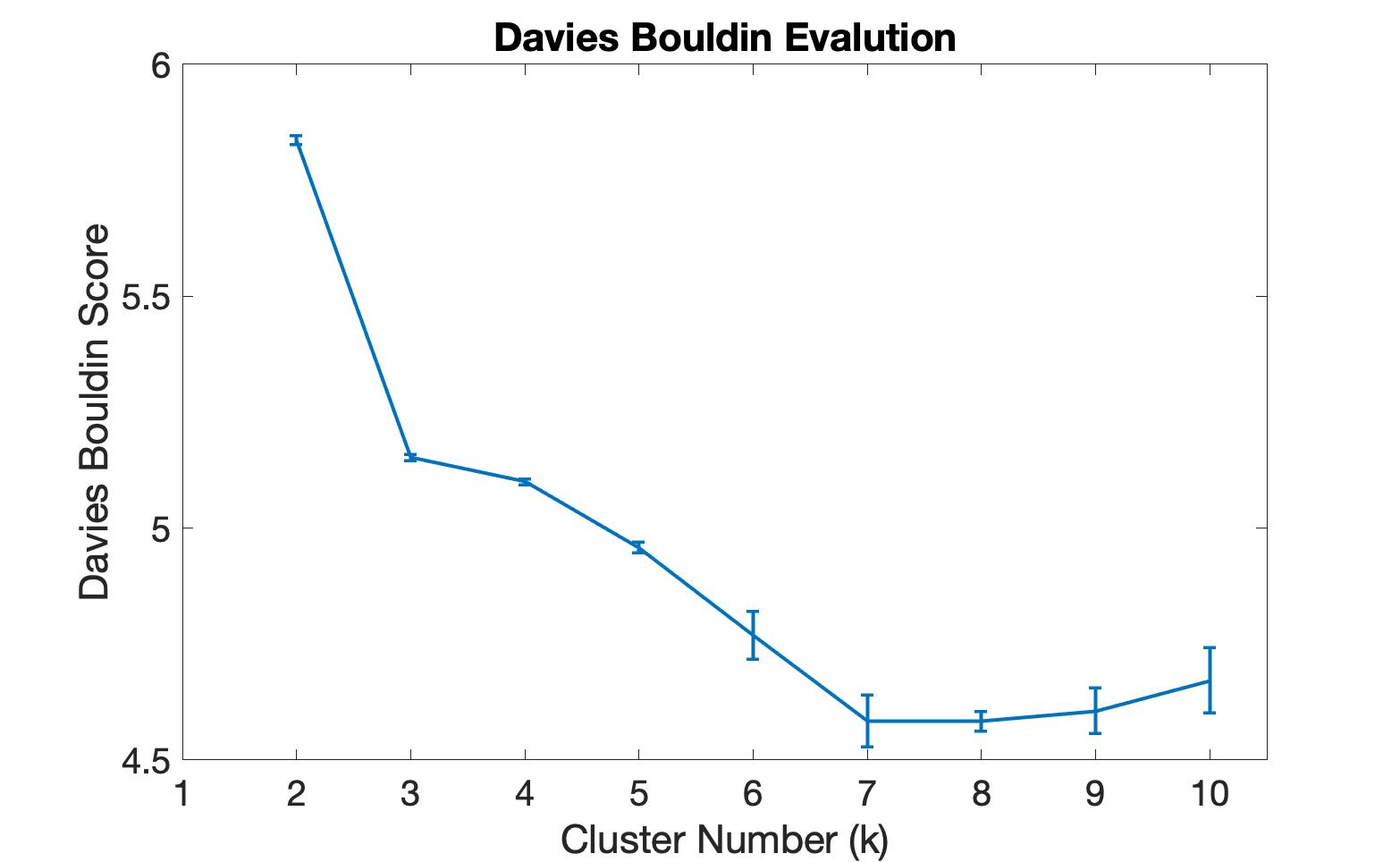

Supplement: Supplementary Figure 1 — Davies–Bouldin plot to choose the optimal number of clusters for a window size of 30 s at Tr = 0.6 s. [file Image_1.TIFF]

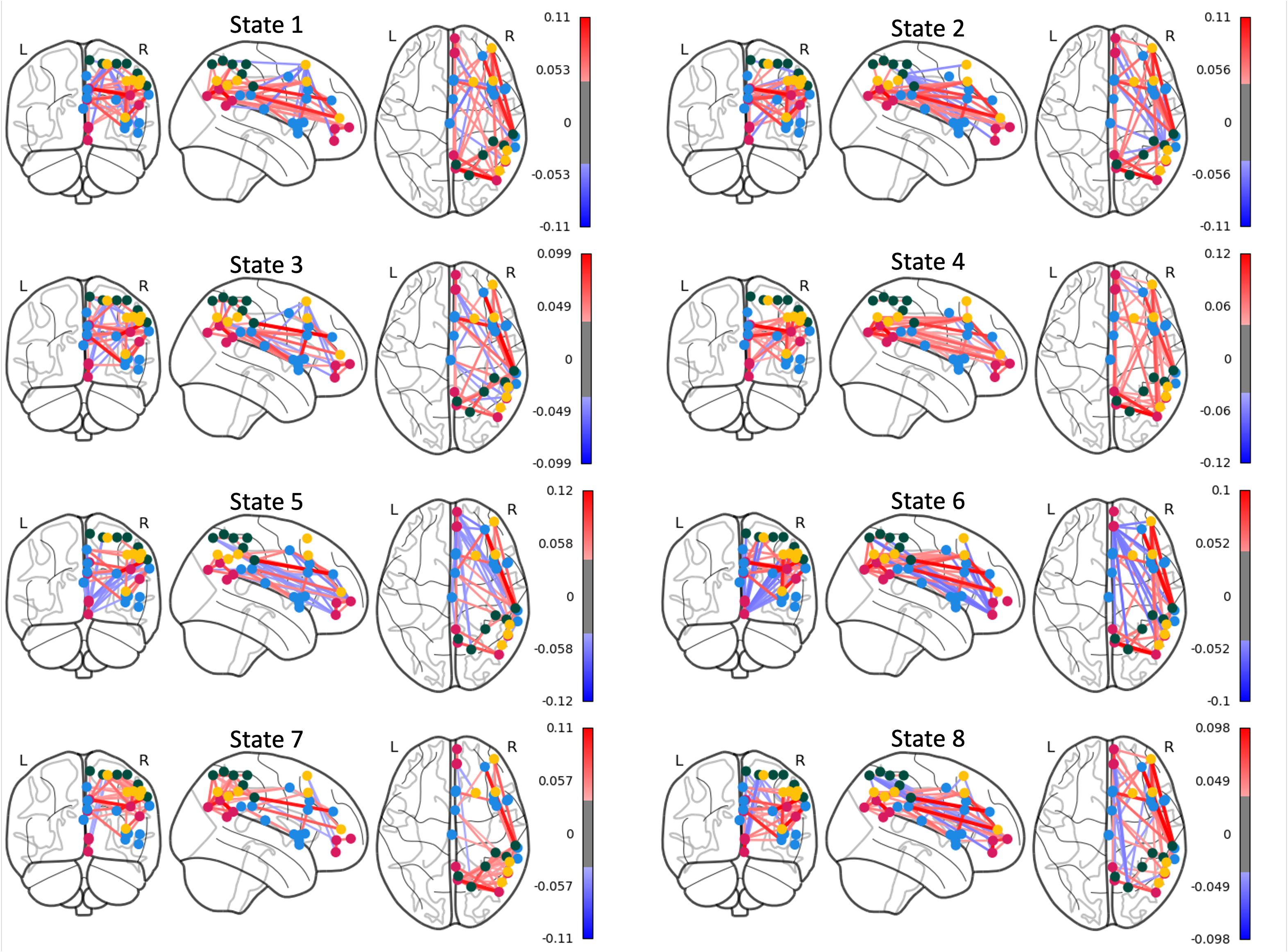

Supplement: Supplementary Figure 2 — Three-plane glass brain view of the eight brain states identified in this work. Red connections indicate positive correlations and blue connections indicate negative correlations. [file Image_2.TIFF]

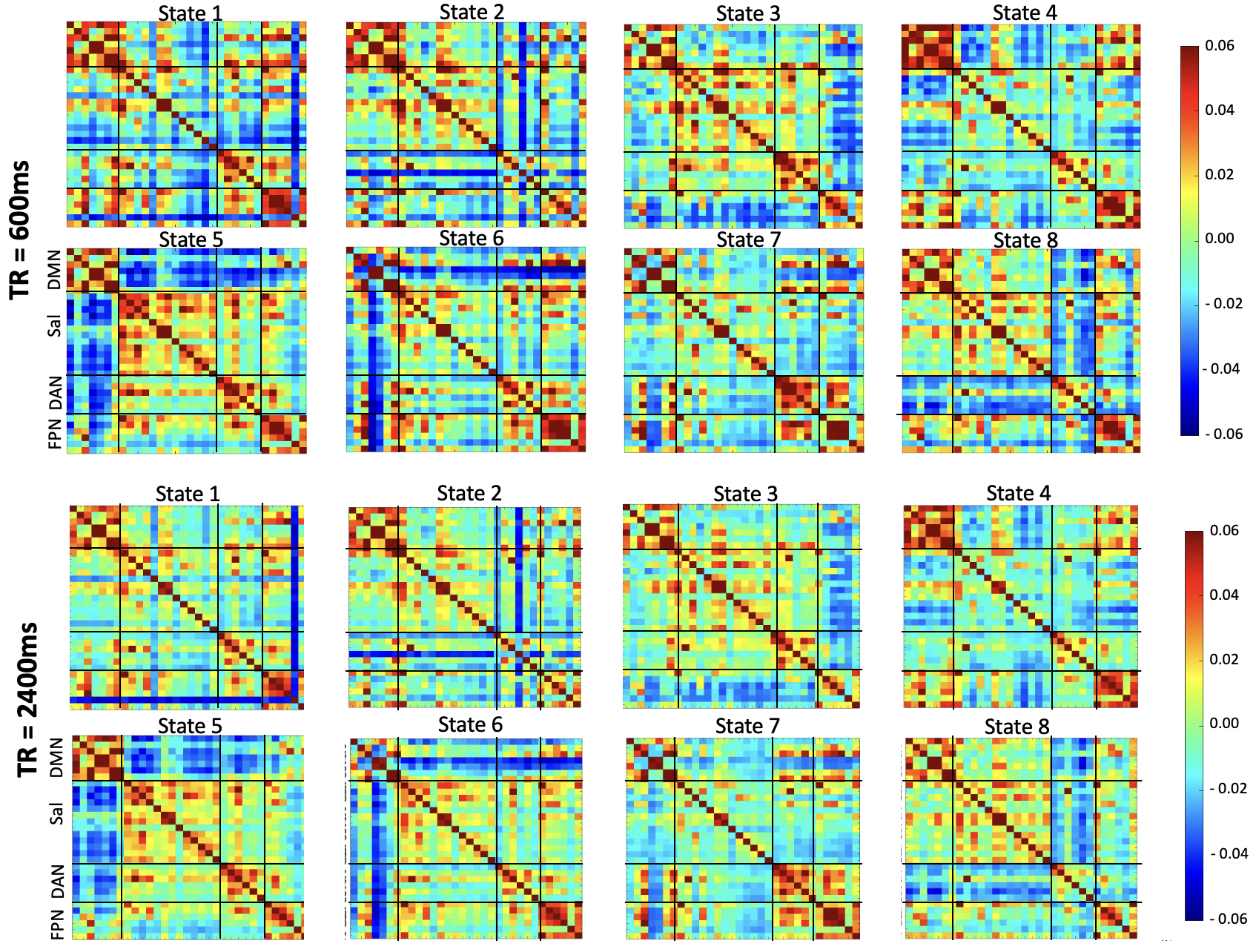

Supplement: Supplementary Figure 3 — Connectivity matrices corresponding to the 8 states identified using TR = 0.6 s and using the same data but subsampled at TR = 2.4 s. [file Image_3.TIFF]

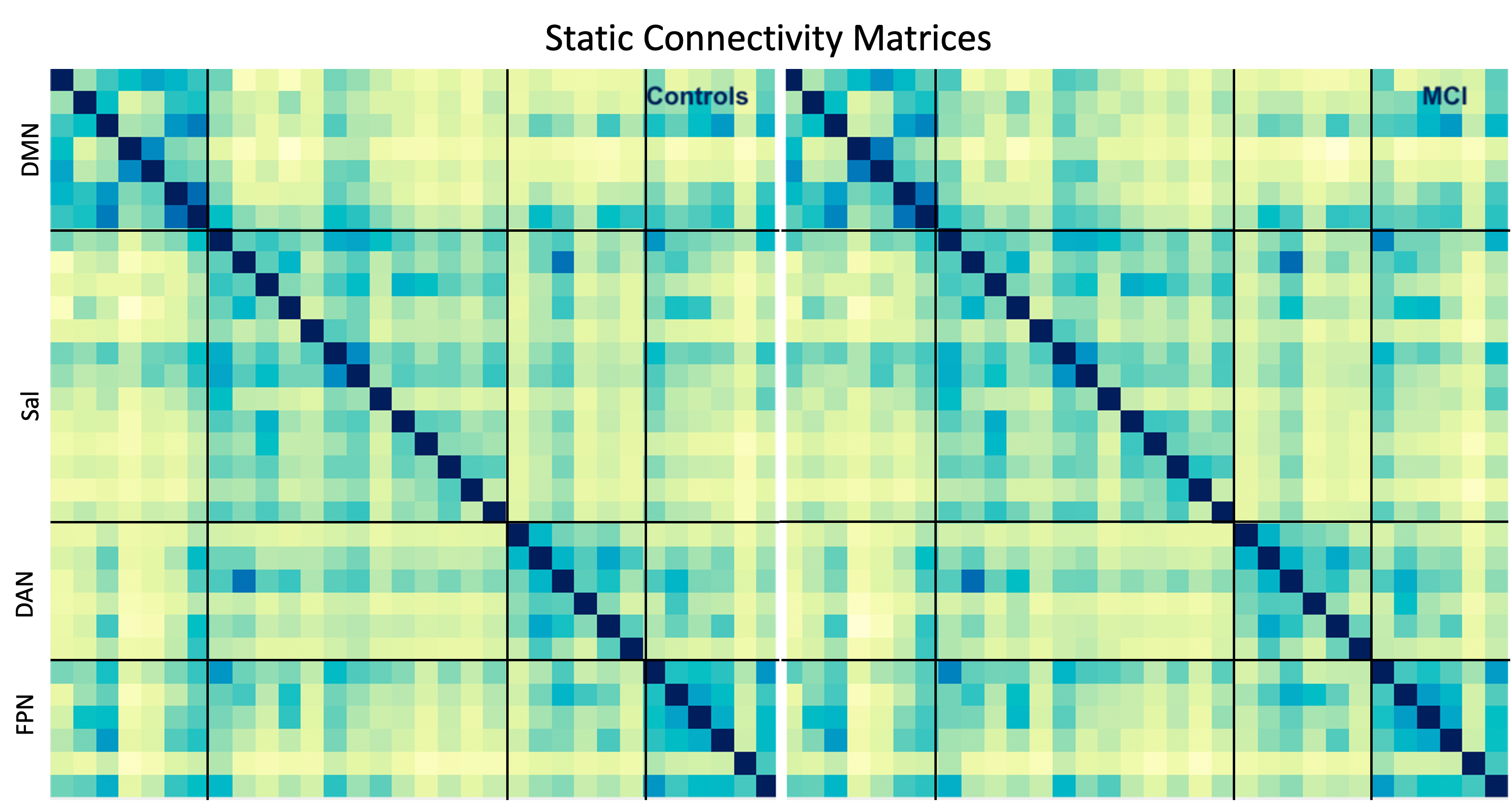

Supplement: Supplementary Figure 4 — Groupwise static connectivity between the same regions as the regions of the DMN, Sal, DAN, and FPN used for dynamic functional connectivity. No significant difference was observed between groups. [file Image_4.TIFF]
